# Reflections on treatment of COVID-19 with traditional Chinese medicine

**DOI:** 10.1186/s13020-020-00375-1

**Published:** 2020-09-03

**Authors:** Hua Luo, Yan Gao, Jian Zou, Siyuan Zhang, Hanbin Chen, Qiao Liu, Dechao Tan, Yan Han, Yonghua Zhao, Shengpeng Wang

**Affiliations:** Institute of Chinese Medical Sciences, State Key Laboratory of Quality Research in Chinese Medicine, University of Macau, Macao SAR, China

**Keywords:** COVID-19, Coronavirus, Traditional Chinese medicine, Prescriptions

## Abstract

Coronavirus disease 2019 (COVID-19) pandemic is spreading rapidly around the globe. By the establishment of an integrative system combining both traditional Chinese medicine (TCM) and western medicine, China has achieved good clinical efficacy in the prevention and control of the pandemic. The advantages of TCM in the treatment of COVID-19 include effective relief of symptoms, retarding the development from mild and moderate to severe, improvement of cure rate, reducing death rates, and promotion of rehabilitation. Besides, according to the different severity levels of individual cases, the National Health Commission of the People’s Republic of China issued treatment guidelines that provide corresponding prescriptions for patients. From the perspective of TCM, this review aims to analyze the role of a variety of TCM prescriptions in the treatment of COVID-19, focusing on the analysis of the “Three TCM prescriptions and three medicines” recommended by the Chinese authorities during the pandemic. We expect that this review will provide insights into the prevention and treatment of COVID-19 with TCM.

## Background

Coronavirus disease 2019 (COVID‑19) belongs to “plague” in the concept of traditional Chinese medicine (TCM), which refers to the virulent infectious diseases with the etiology of epidemic factors [1]. In Chinese history, TCM has battled against hundreds of epidemic/pandemic events, which accumulated a store of effective therapeutic experiences and methods. For instance, in the Song dynasty (960-1279 AD), Chinese practitioners fought against smallpox by variolation. During the battle against SARS in 2003, China leveraged the advantages of TCM and achieved excellent curative outcomes by integrating TCM and western medicine. In the treatment of COVID-19, TCM have also showed favorable effects in amelioration of pathological evolution, and the high cure rate and low mortality in China were highly appreciated by the World Health Organization (WHO).

On Jan 22, 2020, the National Health Commission (NHC) of the People’s Republic of China firstly recommended TCM for the priority treatment of COVID-19 and included four syndromes and corresponding formulae in the Diagnosis and Treatment Protocol for COVID-19 (Trial Version 3). Subsequently, in Trial Version 4 and 5, the prototype of Huashi Baidu Formula (化濕敗毒方) was gradually developed for treating the syndrome of pestilent toxin blocking lung (疫毒閉肺), and got mature in Trial Version 6 and 7. Simultaneously, the ingredients of Xuanfei Baidu Formula (宣肺敗毒方) were applied to treat the syndrome of lung with dampness toxin retention (濕毒鬱肺). Based on numerous clinical therapeutic evidence, NHC published the composition of Qingfei Paidu Decoction (清肺排毒湯) in Trial Version 7, which was used in the treatment of COVID-19 patients from mild to critical stages.

Additionally, since Trial Version 4, NHC recommended Jinhua Qinggan Granule (金花清感顆粒) and Lianhua Qingwen Capsule (連花清瘟膠囊) for attenuating fever of COVID-19 patients during the period of clinical observation, as well as Xuebijing Injection (血必淨注射液) for treating patients in moderate and critical pathological periods. Eventually, Jinhua Qinggan Granule, Lianhua Qingwen Capsule, Xuebijing Injection, Qingfei Paidu Decoction, Huashi Baidu Formula, and Xuanfei Baidu Formula were collectively referred to as “Three TCM prescriptions and three medicines”, becoming a group of effective therapeutics against COVID-19 in China. In this review, the role of a variety of TCM prescriptions for the treatment of COVID-19 are analyzed, aiming to provide insights into the prevention and treatment of COVID-19 with TCM.

## The understanding of TCM on the pathological evolution of COVID-19

### The characteristics of TCM therapy

TCM originated in China and is a traditional medicine that has been used in China for thousands of years [2, 3]. Differentiating from the reductionism of western medicine, the theoretical system of TCM is based on philosophical thinking, derived from the observation and summary of natural materials and their properties and clinical effects, emphasizing the combination of a holistic view, balanced view, and contradiction of *Yin* and *Yang* view [4]. According to the theoretical system of TCM, the material basis of the human body is named as *essence* (精), *qi* (氣), *spirit* (神), *blood* (血) and *body fluids* (津液). The body viscera and its manifestation is named as *zangxiang* (臟象), and the structures that hold the physical basis of the body and connect the viscera are called *meridians and collaterals* (經絡), *vessel* (脈) and *tri*-*jiao* (三焦). Taking the human body as a whole, the body function is in a delicate and dynamic balance. When the homeostasis is broken and the regulatory ability is out of control, the diseases occur.

In TCM system, there are multiple treatment modalities to intervene with a disease, such as acupuncture, moxibustion, and TCM formulae (prescriptions). Prescription is developed based on the theory of TCM syndrome differentiation, which is a medical intervention to restore homeostasis by combining natural materia medica (mostly herbs) in response to specific pathogenesis and pathological changes of the body. In the theoretical system of TCM, herbs are endowed with basic characteristics based on natural properties and clinical effects, such as four properties (hot, cold, warm and cool) and five flavors (sour, sweet, bitter, pungent and salty), which are derived from the real taste of the herbs themselves as well as their functional and clinical effects.

Besides, the TCM theoretical system is comprised of channel tropism (歸經), the ascending and descending, floating and sinking (升降浮沈) established by the guidance of herbs, the toxicity established by herbs’ own toxic and side effects, and the unique properties of each herb are defined. The differentiation of syndrome, based on the TCM theoretical system, focusing on the specific pathogenesis, pathological state and essence of patients, is to form the corresponding treatment ideas, and to use different herb combinations for intervention according to the formulae regular. The differentiation of clinical syndrome fully reflects the characteristics of individualized treatment, which also guide Chinese practitioners to utilize herbs more accurately. Differentiating from the symptoms of western medicine, such as a change in specific pathological manifestation, syndrome of TCM embodies the summary of pathogenesis essence during certain pathological progress of disease. Due to complicated pathogenesis, a single herb often fails to meet the clinical needs and get a desired effect. Therefore, herbs should be combined for comprehensive effects [5]. On the other hand, based on the property of herbs and combination principles, herbs with different properties and combinations are used to achieve specific clinical therapeutic effects and/or reduce toxic and side effects. Moreover, in a TCM prescription, a herb is selected for ameliorating the primary symptoms, which is called sovereign medicinal.

Since the inception of the basic theories of TCM brought forward by the *Yellow Emperor’s Inner Classic* (*Huangdi Neijing*, 黃帝內經), the theoretical system of TCM has gradually evolved, developed, updated and matured, and has eventually formed a synthesized structure of theories with historical background. The principle of herb combination keeps innovating, and the ancient monographs recording various prescriptions are abundant. Presently, it is very important that how to inherit the essence of TCM and provide patients with efficient treatments using traditional clinical experiences and prescriptions.

### The pathogenesis and pathology of COVID-19 in TCM

Being highly infectious and involving similar symptoms during the onset of disease, COVID-19 can be classified as plague (瘟疫) according to the theoretical system of TCM [6]. Usually, plague is not caused by common pathogenic factors (e.g. wind, cold, heat, humidity, dryness and fire), but by some special epidemic toxins. According to the record in Diagnosis and Treatment Protocol for COVID-19 (Trial Version 3), considering the humid weather from November to December in Wuhan, COVID-19 was believed to be associated with dampness and toxin pathogens. Therefore, the initial syndrome was differentiated into dampness obstructing lung (濕邪鬱肺). In Trial Version 4 and 5 of Diagnosis and Treatment Protocol for COVID-19, the first syndrome was cold dampness obstructing lung (寒濕鬱肺), which emphasized the significance of cold pathogen and defined the disease as cold-dampness pestilence [3]. Simultaneously, TCM medicines such as Jinhua Qinggan Granule and Lianhua Qingwen Capsule began to be recommended in the observation period of COVID-19. From Trial Version 6 and 7 of Diagnosis and Treatment Protocol for COVID-19, syndrome of lung with dampness heat retention (濕熱蘊肺) emerged in mild stages, suggesting the heat pathogen was also emphasized. Moreover, Qingfei Paidu Decoction with effects in dispelling exterior pathogen, clearing endogenous heat and drying dampness was recommended for intervening different pathological status. Studies indicate that COVID-19 results from damp toxin, disharmony between qi and body fluid, or combination of drying, moistening and toxin [7]. Currently, it has been generally accepted that the pathogenesis of COVID-19 is the interactions of dampness, toxin, heat, and stasis.

Though some infected people have no obvious symptoms, most patients have common symptoms including low fever, dry cough, chest discomfort, nausea, diarrhea, and even multiple organ failure. According to the clinical manifestation, the pathology of COVID-19 was classified into mild, moderate, severe, and critical stages in Trial Version 6 of Diagnosis and Treatment Protocol for COVID-19. Most of patients in mild-period have symptoms including low fever, fatigue, dry cough, myalgia, nausea or diarrhea. Therefore, TCM mainly adopts an eliminating exterior pathogens approach. Along with the increase in dampness and toxin combining with heat or cold pathogen entering into the interior body, the syndrome presents as lung with dampness toxin retention (濕毒鬱肺) or cold dampness blocking lung (寒濕阻肺), which are combined by heat, toxin, dampness, and cold, accounting for the majority of the symptoms. The clinical symptoms of patients include fever, dyspnea, expectoration, constipation and diarrhea. Therapeutics of eliminating heat and dampness or invigorating spleen to eliminate dampness, as well as detoxification would be used.

At the severe stage, pestilent toxin further attacks lung and reaches *qi* and nutrient aspects, and symptoms begin to exacerbate, such as high fever, dyspnea, shortness of breath, and even vomiting blood and unconsciousness. At this stage, apart from the treatment strategies of eliminating heat and dampness and detoxification, the disfunction of lung and heart need to be regulated by ventilating lung *qi* and removing heat from the heart to restore consciousness by using Huashi Baidu Formula (化濕敗毒方) or Qingying decoction (清營湯). At the critical stage, epidemic toxin has seriously blocked visceral function and resulted in the collapse of *Yang qi* in the human body. The patients present the symptoms with multiple organ functional failure and shock. TCM formulae and medicines with the effects of recuperating collapsed *Yang qi* and restoring consciousness, such as Shenfu decoction (參附湯), Angong Niuhuang pill (安宮牛黃丸), Xuebijing injection (血必淨注射液) and Xingnaojing injection (醒腦靜注射液) are recommended.

**Fig. 1:**
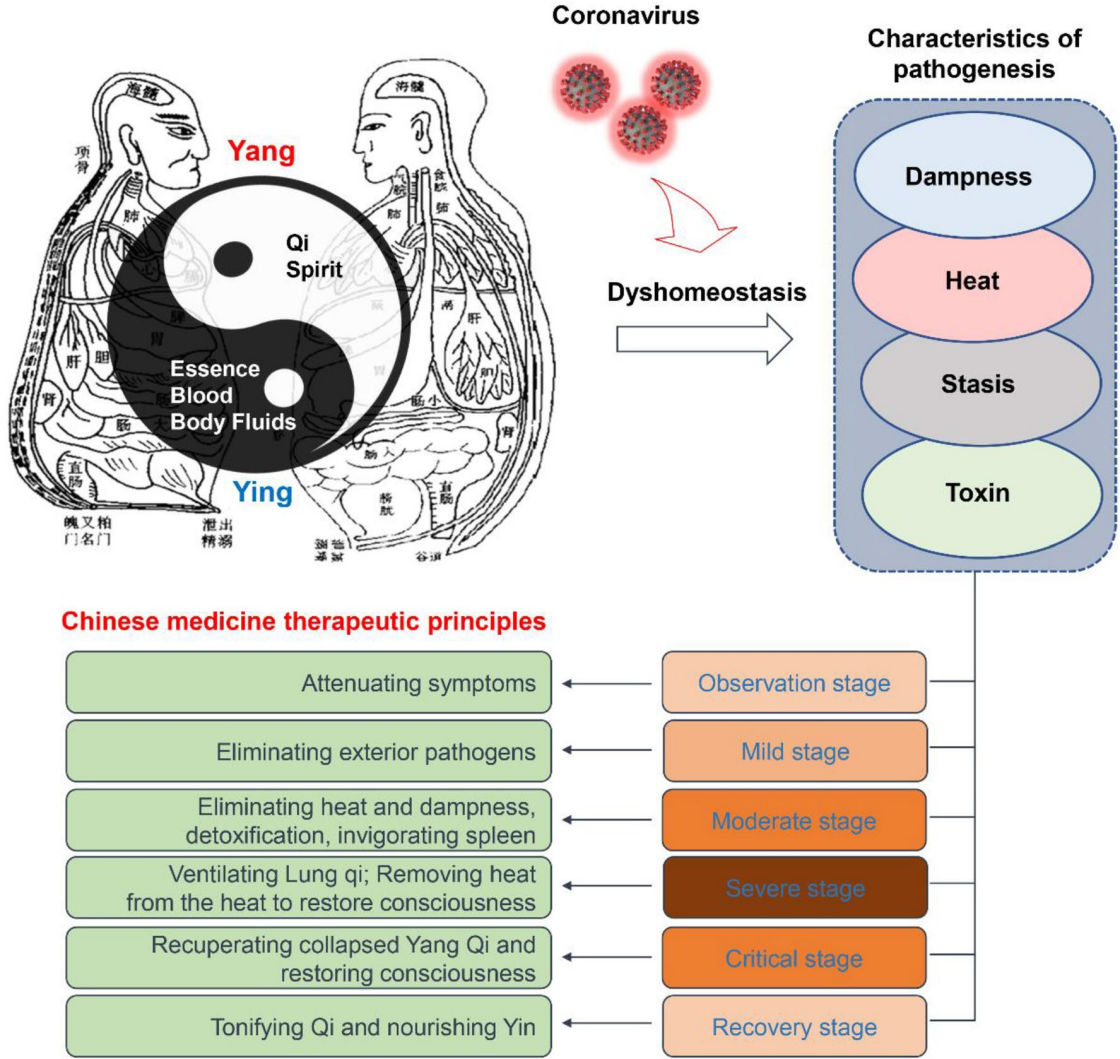
Treatment of COVID-19 with TCM at different stages

After appropriate treatment and the dispelling of epidemic toxins, patients move on to the recovery stage. As *qi* and *Yin* of the patients’ body are deteriorated, TCM formulae with the function of tonifying *qi* and nourishing *Yin* for lung and spleen will be prescribed (Fig. 1).

The natural environment in different regions varies, which diversifies the pathogenesis of diseases and the treatment approaches. For example, Guangzhou province in southern China has a humid and hot climate with a higher degree of dampness. Therefore, Chinese medicines with eliminating dampness effects should be frequently used. However, in Gansu province with dry climate, it is suggested that patients also presented the characteristic of dampness pathogen similar to that in Wuhan [7]. Therefore, the pathogen of dampness toxin should be emphasized and the therapy of eliminating dampness and toxin should be the primary therapeutic strategy against COVID-19.

## Application of TCM for treatment of COVID-19

Up to now, China has made important progress in the fight against the pandemic and TCM has played an crucial role in the treatment of COVID-19. Since the outbreak, the NHC has successively issued seven versions of Diagnosis and Treatment Protocol for COVID-19. In Trial Version 3, TCM therapy began to be recommended. In these therapeutic protocols, three TCM prescriptions and three medicines were selected based on the clinical experience of pandemic prevention and control. They are effective prescriptions with a combination of Chinese and western medicines [8]. Tables 1 and 2 summarized the herbs and prescriptions for COVID-19 treatment at different stages.

**Table 1:** Representative Chinese medical herbs used for prevention and treatment of COVID-19 at different stages

Stage of disease	Herbs
Medical observation	Lonicerae Japonicae Flos, Pogostemonis Herba, Saposhnikoviae Radix, Glycyrrhizae Radix et Rhizoma, Astragali Radix, Atractylodis Macrocephalae Rhizoma, Poria, Citri Reticulatae Pericarpium, Pinelliae Rhizoma, Bupleuri Radix
Mild case Moderate Case Severe Case Critical Case	Magnoliae Officinalis Cortex, Glycyrrhizae Radix Et Rhizoma, Pogostemonis Herba, Ephedrae Herba, Armeniacae Semen Amarum, Citri Reticulatae Pericarpium, Poria, Amomi Fructus, Platycodonis Radix, Zingiberis Rhizoma Recens
	Ephedrae Herba, Armeniacae Semen Amarum, Scutellariae Radix, Coicis Semen, Glycyrrhizae Radix et Rhizoma Tsaoko Fructus, Pogostemonis Herba, Arecaesemen, Magnoliae Officinalis Cortex, Arecaesemen
	Ephedrae Herba, Arecaesemen, Armeniacae Semen Amarum, Glycyrrhizae Radix et Rhizoma Descurainiae Semen Lepidii Semen, Rhei Radix et Rhizoma, Scutellariae Radix, Persicae Semen, Paeoniaeradix Rubra, Arecaesemen
	Ginseng Radix et Rhizoma, Aconm Lateralis Radix Praeparaia, Corni Fructus, Ophiopogonis Radix, Glycyrrhizae Radix et Rhizoma, Schisandrae Chinensis Fructus, Zingiberis Rhizoma, Ephedrae Herba, Asari Radix et Rhizoma, Arecaesemen
Convalescence	Glycyrrhizae Radix et Rhizoma, Pinelliae Rhizoma, Poria, Ophiopogonis Radix, Atractylodis Macrocephalae Rhizoma, Citri Reticulatae Pericarpium, Astragali Radix, Codonopsis Radix, Amomi Fructus, Pseudostellariae Radix

**Table 2:** Representative Chinese patent drugs used for treatment of COVID-19 at different stages

Stage of disease	TCM formula
Medical observation	Sanao tablet, Yupingfeng powder, Lingyang Ganmao tablet, Fangfeng Tongsheng pill, Huoxiang Zhengqi capsule, Kangbingdu oral liquid, Shenge Yifei capsule, Fufang Yinchai granule, Lianhua Qingwen capsule, Jinhua Qinggan granule, Qingkailing soft capsule, Shanlameiye granule, Shenling Baizhu capsule, Shufeng Jiedu capsule, Qixiang Yiqi Jiedu granule
Mild case	Jingbai Fangdu powder, Jiuwei Qianghuo pill, Lanqin oral liquid, Tanreqing capsule, Ertong Kanggan granule, Chaishi Tuire Granule, Jinhua Qinggan granule, Kangbingdu oral liquid, Toujie Quwen granule, Shufeng Jiedu capsule, Kegan Liyan oral liquid, Qingre Huashi oral liquid, Xiaoer Qingre Lifei oral liquid
Severe case	Angong Niuhuang pill, Xiaochaihu granule, Fangfeng Tongsheng pill, Huashi Baidu decoction, Xiyanping injection, Xingnaojing injection, Reduning injection, Tanreqing injection, Xuebijing injection
Critical case	Zhibao pill, Zixue pill, Suhexiang pill, Shenmai injection, Shengmai injection, Angong Niuhuang pill, Shenfu injection, Xingnaojing injection, Reduning injection, Tanreqing injection, Xuebijing injection
Convalescence	Shenling Baizhu capsule

Jinhua Qinggan Granule is a Chinese medicine developed during the 2009 H1N1 influenza pandemic [9]. It consists of 12 herbs including Jinyinhua (Lonicerae Japonicae Flos), Shigao (Gypsum Fibrosum), Mahuang (Ephedrae Herba), Kuxingren (Armeniacae Semen Amarum), Huangqin (Scutellariae Radix), Lianqiao (Forsythiae Fructus), Zhebeimu (Fritillariae Thunbergii Bulbus), Zhimu (Anemarrhenae Rhizoma), Niubangzi (Arctii Fructus), Qinghao (Artemisiae Annuae Herba), Bohe (Menthae Haplocalycis Herba) and Gancao (Glycyrrhizae Radix et Rhizoma). The traditional functions of this medicine are dispersing wind, ventilating the lung, clearing heat, and removing toxicity [7]. Fundamental research and the clinical study showed that Jinhua Qinggan Granule had promissing effect on H1N1 influenza and excellent developmental value [10]. Results from a double-blind,randomized,controlled trial showed that it could safely and effectively treat influenza with syndromes of wind-heat attacking the lung [11]. Moreover, it can effectively reduce the serum levels of various cytokines and enhance the immune function of patients with influenza [7]. During the fight against COVID-19, Jinhua Qinggan Granule was selected in the Diagnosis and Treatment Protocol for COVID-19 since the Trial Version 4. Clinical practice has demonstrated that it has a significant therapeutic effect on the clinical symptoms of mild COVID-19 patients, such as fever, cough, fatigue, and expectoration, and can relieve the psychological anxiety of patients [12]. Network pharmacology and molecular docking techniques revealed that the action mechanism might be attributed to its active constituents such as kaempferol, baicalein and oroxylin A, which regulate multiple signaling pathways by binding with SARS-CoV-2 3CL hydrolase and acting on targets such as ACE2, PTGS2, BCL2, CASP3, HSP90AB1, HSP90AA1, PTGS1 and NCOA2 [13, 14].

Lianhua Qingwen Capsule is a TCM preparation composed of Lianqiao (Forsythiae Fructus), Jinyinhua (Lonicerae Japonicae Flos), Zhi Mahuang (Processed Ephedrae Herba), Mianmaguanzhong (Dryopteridis Crassirhizomatis Rhizoma), Ban Lan Gen (Isatis Radix), Shigao (Gypsum Fibrosum), Bohenao (Menthol), Guanghuoxiang (Pogostemonis Herba), Hongjingtian (Rhodiolae Crenulatae Radix et Rhizoma), Yuxingcao (Houttuyniae Herba), Dahuang (Rhei Radix et Rhizoma), Chao Kuxingren (Processed Armeniacae Semen Amarum) and Gancao (Glycyrrhizae Radix et Rhizoma). Lianhua Qingwen Capsule exhibits broad-spectrum antiviral effects in clinical studies [15, 16]. Its therapeutic effects are generally better than that of cefalexin capsules [17]. Lianhua Qingwen Capsule can not only resist the virus but also has a significant antibacterial and anti-inflammatory effect, so as to improve the symptoms of influenza and accelerate the dissipation of bronchial pneumonia [18]. Results of the acute toxicity study showed that it was safe under conventional clinical doses. Therefore, Lianhua Qingwen Capsule have been used in the treatment of children with exogenous high fever. Moreover, it played an important role in the treatment of critical cases and severe cases of H1N1 in children, and showed higher safety than the antiviral drug oseltamivir [19]. In clinical practices, Lianhua Qingwen Capsule also achieved a good therapeutic effect in combination with other drugs, such as Tanreqing injection, potassium dehydroandrograpolide succinate injection, and azithromycin injection in the treatment of H1N1 [20]. Besides, the combination of ribavirin and vitamin C also significantly improved the therapeutic efficacy during the treatment of viral upper respiratory infection [21]. In the treatment for COVID-19, the results suggested that Lianhua Qingwen Capsule not only attenuated the SARS-COV-2 replication in vitro but also improved clinical symptoms of patients with COVID-19 and increased clinical cure rate [22, 23].

Xuebijing Injection is extracted from Honghua (Carthami Flos), Chishao (Paeoniae Radix Rubra), Chuanxiong (Chuanxiong Rhizoma), Danshen (Salviae miltiorrhizae Radix et Rhizoma) and Danggui (Angelicae Sinensis Radix). Its main components include safflor yellow A, ligustrazine, ferulic acid, salvianic acid A, paeoniflorin, and protocatechualdehyde. It is effective in removing blood stasis and toxin and has been widely used in the systemic inflammatory response syndrome induced by infection [22]. In animal studies, Xuebijing Injection could significantly reduce the level of serum endotoxin of septic rats. In clinical studies, the combination of Xuebijing Injection and western medicine rescue measures could regulate the immune function of patients and improve the condition of sepsis. Besides, when combined with blood purification, Xubijing also played a role in adjusting immunity in the treatment of sepsis [23]. The standard treatment of sepsis supplemented by Xuebijing Injection significantly increased the recovery rate of the treatment [24]. Based on the network of “drug target pathway”, over 20 compounds of Xuebijing Injection participated in the regulation of 550 targets and 10 inflammation and immune-related pathways [25]. Xubijing Injection has also played an important role in the clinical fight against COVID-19. Network pharmacology revealed that the possible mechanism of action might be associated with the regulation of 70 proteins that interact with ACE2, thereby relieving the inflammatory response and inhibiting the replication of the virus [26].

Qingfei Paidu Decoction consists of four classic prescriptions from the Treatise on Exogenous Febrile and Miscellaneous Diseases (Shanghan Zabing Lun, 210 AD), including Maxing Shigan Decoction, Shegan Mahuang Decoction, Xiao Cai Hu Decoction, and Wuling powder. Qingfei Paidu Decoction is composed of 21 herbs, including Mahuang (Ephedrae Herba), Zhi Gancao (Processed Glycyrrhizae Radix et Rhizoma), Xingren (Armeniacae Semen), Sheng Shigao (Gypsum Fibrosum), Guizhi (Cinnamomi Ramulus), Zexie (Alismatis Rhizoma), Zhuling (Polyporus), Baizhu (Atractylodis Macrocephalae Rhizoma), Fuling (Poria), Chaihu (Bupleuri Radix), Huangqin (Scutellariae Radix), Jiang Banxia (Pinelliae Rhizoma Praeparatum Cum Zingibere et Alumine), Shengjiang (Zingiberis Rhizoma Recens), Ziwan (Asteris Radix), Donghua (Farfarae Flos), Shegan (Belamcandae Rhizoma), Xixin (Asari Radix et Rhizoma), Shanyao (Dioscoreae Rhizoma), Zhishi (Aurantii Fructus Immaturus), Chenpi (Citri reticulatae Pericarpium) and Huoxiang (Pogostemonis Herba) [27, 28]. As a generic prescription recommended in the Trial Version 6 and 7 of Diagnosis and Treatment Protocol for COVID-19, Qingfei Paidu Decoction has shown remarkable curative effects in clinical treatment [29–32]. Given the individual differences of patients, Qingfei Paidu Decoction should be personalized appropriately during clinical application [31]. Clinically, Qingfei Paidu Decoction is also used in combination with western medicine to treat COVID-19, such as with lopinavir and ritonavir tablets, methylprednisolone sodium succinate injection, moxifloxacin hydrochloride, and sodium chloride injection, interferon α2b injection to treat severe cases of COVID-19 [33]. When used in combination with the Kaletra, this medicine can improve the pulmonary conditions of patients, evidenced by the lung images [34]. Qingfei Paidu Decoction also posesses anti-inflammatory and antiviral effects [35]. Network pharmacology study found that the first three active components of Qingfei Paidu Decoction in the treatment of COVID-19 were quercetin, luteolin and kaempferol, which could regulate various targets including AKT1, JUN, MAPKs, IL-6, RELA, STAT1 to inhibit the inflammatory response, modulate immune function, reduce lung injury, and protect nerve function [28, 36].

Huashi Baidu Prescription is a recommended prescription for severe epidemic toxin blocking the lung syndrome in the Trial Version 6 and 7 of Diagnosis and Treatment Protocol for COVID-19. This prescription contains experts’ wisdom and the characteristics of COVID-19 [37]. It consists of 14 herbs, including Sheng Mahuang (Ephedrae Herba), Huoxiang (Pogostemonis Herba), Sheng Shigao (Gypsum Fibrosum), Xingren (Armeniacae Semen), Fa Banxia (Pinellinae Rhizoma Praeparatum), Houpo (Magnoliae officinalis Cortex), Cangzhu (Atractylodis Rhizoma), Caoguo (Tsaoko Fructus), Fuling (Poria), Sheng Huangqi (Astragali Radix), Chishao (Paeoniae Radix Rubra), Tinglizi (Lepidii/Descurainiae Semen), Sheng Dahuang(Rhei Radix et Rhizoma) and Gancao (Glycyrrhizae Radix) [37, 38]. This prescription exhibited effects via multi-link comprehensive treatment for COVID-19 and significantly shortened the time of recovery (meaning, nucleic acid test turning negative), hospital stay, and improved clinical symptoms including physical and chemical examinations and CT results of the lung. The liver and kidney function of patients treated with Huashi Baidu Formula were examined and no adverse reactions were found. At present, this medicine has been approved for a clinical trial by the National Medical Products Administration.

Xuanfei Baidu Formula is a TCM prescription in the first-line treatment proposed by CAS Academician Boli Zhang and Professor Qingquan Liu for damp-toxin retention in lung-syndrome patients. The formula consists of 13 herbs, including Sheng Mahuang (Ephedrae Herba), Kuxingren (Armeniacae Semen Amarum), Sheng Shigao (Gypsum Fibrosum), Sheng Yiyiren (Coicis Semen), Maocangzhu (Atractylodis Rhizoma), Guanghuoxiang (Pogostemonis Herba), Qinghao (Artemisiae Annuae Herba), Huzhang (Polygoni cuspidati Rhizoma eT Radix), Mabiancao (Verbenae Herba), Lugen (Phragmitis Rhizoma), Tinglizi (Lepidii/Descurainiae Semen), Huajuhong (Citri Grandis Exocarpium rubrum) and Gancao (Glycyrrhizae Radix et Rhizoma). Network pharmacology study found regulation of inflammation factors such as IL-6, chemokines CXCL8, etc., and related T cells (Th17, Th1, Th2) by Xuanfei Baidu Decoction suppresses the inflammatory storms and excessive activation of immune response after SARS-CoV-2 infection [8]. It is important to reduce inflammation during the early stage of virus infection,which may therefore enhance immunity during the late period. This bidirectional regulation showed improvements in the symptoms associated with SARS-CoV-2 infection. The important targets of the Xuanfei Baidu Decoction were mainly concentrated in the pathways related to viral infection and lung injury. To some extent, this explains the remarkable efficacy of this prescription in accelerating the recovery of moderate cases and preventing moderate cases from developing into severe cases.

Now the “Three TCM prescriptions and three medicines” in the fight against COVID-19 have exhibited significant curative effects in clinical applications. Statistical analysis revealed that five TCM herbs were applied most frequently in these formulations, including Guanghuoxiang (Pogostemonis Herba), Gancao (Glycyrrhizae Radix et Rhizoma), Shigao (Gypsum Fibrosum), Kuxingren (Armeniacae Semen Amarum) and Mahuang (Ephedrae Herba). Therefore, the actions of antiviral, immune regulatory, and amelioration of symptoms of these five medicines of the “Three TCM prescriptions and three medicines” are systemically summarized and reviewed.

### Antiviral effect

Guanghuoxiang (*Pogostemon cablin* (Blanco) Benth.) is a plant belonging to the family Lamiaceae, originally from southeast Asian countries. After being introduced to China, it is mainly produced in Guangdong and the whole herb may be used as medicine [39–41]. Patchouli oil is a volatile oil extracted from *Pogostemon cablin* (Blanco) Benth by steam distillation. The main active components of Patchouli are patchouli alcohol, patchoulene, *α*-guaiene, *δ*-guaiene, *α*-patchoulene, *β*-patchoulene, pogostone, and aristolone. In recent years, extensive studies on the pharmacological effects of patchouli oil have been performed. Patchouli oil acts against coxsackievirus, adenovirus, influenza A virus, and respiratory syncytial virus through in vitro antiviral experiments. At the same time, patchouli alcohol also acts against coxsackievirus, adenovirus, and influenza A virus. These findings provide an important basis for research and development of a new anti-viral drug against viral infections [42, 43]. Similarly, ethyl acetate and methanol extracts of patchouli exhibited good anti-coxsackievirus activity in vitro [44].

Gancao is the dry root and rhizome of Leguminosae plant *Glycyrrhiza uralensis* Fisch., *Glycyrrhiza inflate* Bat. or *Glycyrrhiza glabra* L. from the northeast and northwest China. It also widely distributed in central Asia and southern Europe. This herb is widely known as the “Regulator of the Chinese herbal medicine” [45]. Gancao contains a variety of active ingredients, with the highest content of glycyrrhizic acid, glycyrrhizic flavone, and licorice polysaccharides. Glycyrrhizic acid, also known as glycyrrhizin, exists in the form of sylvite or calcium salts in Gancao [45]. Numerous studies have shown that glycyrrhizic acid has a wide range of antiviral activities. In particular, it showed a significant therapeutic effect against the subtype H9N2 influenza virus [45] and the coronavirus SARS-CoV. Besides, the coronavirus SARS-CoV-2 often targets angiotensin-converting enzyme 2 (ACE2) receptor, and molecular docking technology indicated that glycyrrhizic acid can combine with ACE2, suggesting the potential of glycyrrhizic acid for the treatment of COVID-19 [46]. Meanwhile, glycyrrhizin showed significant antagonistic effects against herpes simplex virus, human immunodeficiency virus (HIV), and hepatitis viruses including hepatitis B virus (HBV), and hepatitis C virus (HCV) [47]. As a metabolite of glycyrrhizin, glycyrrhetinic acid has a good antiviral activity as well, against African lymphocytoma virus and the herpes virus related to kaposi sarcoma [48].

Mahuang (Ephedrae Herba) is the dry grass stem of ephedra shrub from Ephedra sinica Stapf, E. intermedia Schrenk et C.A.Mey. or E. equisetina Bge. It mainly contains alkaloids and other components that vary with species. In a study of anti-influenza A (H1N1) activity of Mahuang decoction in vitro, Mahuang decoction blocked the invasion of influenza virus into host cells to resist its adsorption process, and inhibited the biosynthetic of influenza virus mainly by down-regulating the expression levels of TLR4, TLR7, MyD88 and TRAF6 mRNA in cells, to achieve the anti-influenza effect [49].

### Immunoregulation effect

Researchers established a serum pharmacology testing method for essential oil from patchouli leaves. They investigated the effect of serum containing the medicine on mice’s peripheral leukocytes, peritoneal macrophages, and the spleen lymphocytes at different time points. The results showed that the serum-containing medicine significantly activated these cells, suggesting that essential oil has a certain immunoregulatory role in mice [50]. In the immunologically impaired mice model, patchouli alcohol increased thymus index, spleen index and the content of hemolysin and clearance index, as well as inhibited the shrinkage of spleen and thymus in mice infected with influenza virus by improving systemic immune function [51]. Pogostone showed a significant inhibitory effect on Con-A-induced mice T lymphocytes proliferation and activation in vitro and effectively protected Con-A-induced apoptosis. Therefore, this natural compound exhibited a certain inhibitory effect on adaptive immunity [52].

Licorice polysaccharide is one of the major active ingredients of Gancao, which exhibits immunomodulatory activities. As a polysaccharide, licorice polysaccharide can promote human peripheral blood γδT cells proliferation and secretion of cytokines, thereby killing cancer cells [53]. It can also inhibit allergy-induced sarcoma cells apoptosis and enhance immunity by activating the immune surveillance system [54]. Besides, licorice polysaccharide could also enhance the activity of immune cells and improve the ability of macrophages to engulf pathogenic microorganisms, thus playing an important antiviral role [55].

Amygdalin, also known as vitamin B_17_, is a toxic glycoside compound mainly found in bitter almond, with a content of about 2% to 3%. Amygdalin is an effective component of Kuxingren and is often used as an expectorant and adjuvant anti-cancer drug [56]. Amygdalin plays an important role in the regulation of the immune system. For instance, amygdalin can inhibit the expression of TNF-α and sICAM-1, effectively inhibit joint swelling in joint swelling model rats, and alleviate the pathological damage, thus achieving successful treatment of rheumatoid arthritis (an autoimmune disease) [57]. Asthma is a chronic inflammatory airway disease mainly caused by environmental factors and immune factors. The immune imbalance is a hotspot of research on the mechanism of asthma, among which Th1/Th2 imbalance is an important mechanism as Th1 cells mainly release INF-γ, resisting allergic reactions, whereas Th2 cells mainly release IL-4 that can promote the allergic reaction. A study found that laetrile increased the level of INF-γ and lowered the level of IL-4 in an asthma rat model, thus suppressing the appearance and aggregation of inflammatory mediators to reduce asthma airway inflammation [58]. Moreover, amygdalin can inhibit adjuvant inflammation, enhance the activity of macrophages, improve the phagocytic function of macrophages, and regulate the immune system [59]. It can directly inhibit the proliferation of immune cells, thereby playing an immunosuppressive role [60].

Mahuang (Ephedrae Herba) also plays an important role in regulating immunity. In the study investigating the effect of intragastric administration single dose of Mahuang on the humoral immunity of mice, it was found that Mahuang significantly improved the activity and hair gloss of Kunming mice, suggesting that it increased the humoral immunity [61]. On the other hand, Mahuang could inhibit the cellular immune function of mouse [62], and suppress the spleen index, phagocytic coefficient α of mononuclear macrophages and complement activity of Kunming mice [63]. Similarly, Shegan Mahuang Decoction can alleviate airway inflammation in the rat bronchial model, regulate the immune imbalance of Th1/Th2, and treat bronchial asthma by inhibiting the activation of Th2 cells, suggesting that Shegan Mahuang Decoction has significant potential in the treatment of asthma [64]. By preliminarily exploring the mechanism of Shegan Mahuang Decoction in the treatment of bronchial asthma by network pharmacology, it was found that HPS90AA1, TGFB1, NFKB2, and MMP9 may be the key targets of Shegan Mahuang Decoction in the pathway of immune-inflammatory treatment, and NR3C1 and PGR may be the key targets for the treatment of refractory asthma [65].

The researcher compared the clinical effects and serum cytokine levels of 174 influenza patients on Day 3 and Day 5 after the administration. The results showed that Jinhua Qinggan Granule significantly reduced the levels of various cytokines in serum, enhanced the immune function, and exhibited significant therapeutic effect with a decent safety profile, which demonstrated its clinical use value. However, its efficacy did not increase significantly with the increase of dose. Thus a relatively low, effective dose would be recommended [7].

### Antipyretic effect

Fever Syndrome in TCM theory often refers to a series of comprehensive symptoms, including fever, thirst, red tongue, constipation, restlessness, and quick pulse. Seasonal factors, diet, living/sleeping and other external factors are usually the reasons for the occurrence of illness. COVID-19 is a type of fever disease caused by external factors, with symptoms such as fever, thirst, fatigue and dry cough. Many scientists have found that high fever models can be created in animals by intravenous injection of LPS. Licorice zinc granule significantly reduced the antipyretic time of children with viral enteritis in the treatment [66]. Bai Hu Tang (BHT) is a classic anti-fever formula comprised of Gancao (Glycyrrhizae Radix et Rhizoma), Zhimu (Anemarrhenae Rhizoma), Shigao (Gypsum Fibrosum) and Gengmi (Oryzae Semen). It is traditionally used as an anti-fever treatment to promote body fluid production and relieve thirst. BHT (750 mg dry extract/kg body weight) up-regulated F-actin, coronin, Rac, and MHC I, acting in phagocytosis and cross-presentation, in the high fever model produced in rabbits by LPS injection. These results suggested that BHT might promote pyrogen clearance by promoting antigen phagocytosis, degradation, and cross-presentation in the liver [67]. The antipyretic effect of Shigao might be attributed to the combined action of various trace elements. The indirect antipyretic effect is likely related to the control of infection by iron, copper, selenium, etc. which regulate the immune system [68]. Mahuang Decoction can dispel exterior pathogens, so the patients who show exterior symptoms including an aversion to cold, fever, headache, body pain, no sweat and shortness of breath, are suitable for Mahuang Decoction treatment. However, the sweating effect of this prescription is strong and thus it should only be used under the guidance of qualified physicians [69] Lianhua Qingwen Capsule can reduce the inflammatory reaction, inhibit the mouse’s ear swelling caused by xylene, and increase the permeability of capillaries in the abdominal cavity of mice caused by 0.6% acetic acid. The experiment indicated that Lianhua Qingwen Capsule have a better therapeutic effect on the fever caused by typhoid, paratyphoid A and B, and can reduce fever and inflammatory. A randomized, double-blinded, and multi-center clinical study carried out in 3A hospitals in China showed that the effective rate of Lianhua Qingwen Capsule in the treatment of influenza fever was over 90%. Modern pharmacology has revealed that Lianhua Qingwen Capsule have antipyretic and antiphlogistic activities, as well as functions of resisting common respiratory tract viruses and influenza viruses and regulating immune system. Lianhua Qingwen Capsule have good antiviral, antibacterial, anti-inflammatory, antipyretic, analgesic, phlegm&cough-dissipating, and bidirectional immune-regulatory effects [70]. Some researchers observed the clinical efficacy of Xuebijing injection in the treatment of stroke-associated pneumonia. Patients received an intravenous drip of 50 mL Xuebijing Injection twice a day for 7 days. Compared with the control group, the Xuebijing Injection group significantly reduced the antipyretic and antitussive time [71]. It was found that on the basis of western medicine treatment, Xuebijing Injection can effectively shorten the time of hospitalization and fever, and was beneficial to the control of convulsion in the treatment of children with febrile convulsion [72].

### Relieving cough

As a result of the different etiology and organism reactivity in the respiratory tract, cough and cough-up phlegm are the main symptoms. Cough and cough-up phlegm are mostly accompanied by fever, headache, and aversion to cold. To against cough, an important approach in the treatment of COVID-19 is to use antitussive drugs. In modern pharmacology, the mouse cough model is mainly established with the administration of concentrated ammonia water to evaluate the antitussive effects of drugs. Gancao (Glycyrrhizae Radix et Rhizoma) is an anti-cough expectorant TCM with a long history, highly praised for its medical function of cough-relieving in various Chinese sayings and poems. The activities of 14 major compounds and crude extracts of Gan Cao was evaluated by using the classical ammonia-induced cough model and the phenol red secretion model. It was found that liquiritin apioside, liquiritin, and liquiritigenin significantly reduced the frequency of cough by 30–78% when the administered dose was 50 mg/kg (i.g.). Further research also found that liquiritin apioside and liquiritin are the main antitussive components of Gancao, whose mechanisms were related to the peripheral nerve and central innervation [73]. The water extract of Gan Cao showed an inhibitory effect on the ammonia induced cough in mice [74]. The volatile oil of patchouli could significantly prolong the time of ammonia solution spray in half of the experimental mice (EDT50), indicating that the volatile oil of patchouli had an obvious antitussive effect [75]. Maxing Ganshi decoction (MXGSD), consisting of Mahuang (Ephedrae Herba), Kuxingren (Armeniacae Semen Amarum), Gancao (Glycyrrhizae Radix et Rhizoma) and Shi Gao (Gypsum Fibrosum), is an important TCM prescription for the treatment of cough. On the citric acid-induced cough model, it was found that MXGST extract had an obvious dose-dependent anti-coughing effect on guinea pigs, and its anti-coughing mechanism was related to the partial relaxation of bronchial smooth muscle by blocking acetyl cholinergic and histaminergic receptors [76]. A clinical study found that Maxing Ganshi Jiawei Decoction, in combination with anti-infection western medicine, exhibited shorter average antipyretic and antitussive time than that of the control group of patients treated with anti-infection western medicine alone [77]. By microinjection of citric acid solution into the larynx, a laryngeal cough model of the guinea pig was built. Mahuang extract was dissolved in water and the model animals took it freely for 3 days, the number of coughs in 10 min was subsequently counted and calculated. The results showed that Mahuang extract reduced the frequency of cough [78]. Another study found that ephedrine extract at a dose of 200 mg/kg reduced the cough response to 68.3% of the initial level, but had no significant antitussive effect. When made into a composite antitussive Asgen^®^, ASG-1 510 mg/kg (including EE + SE + CSB + AAP) was more effective than any combinations and any single components in this study [79]. The antitussive effect of Mahuang Decoction made from the powder was better than that of Mahuang Decoction made from pieces [80]. Combining Mahuang (Ephedrae Herba) and Xingren (Armeniacae Semen) in 2: 1 ratio had a better therapeutic effect on relieving cough and asthma than other compatibility programs, and the mechanism might be related to down-regulation of CGRP level and a variety of inflammatory cytokines [81]. Ephedra tannic acid could decrease WAm/Pbm and WAi/Pbm and change airway remodeling. The expression of CACC1 and CACC4 in lung tissues was down-regulated, and the expression of TMEM16A was up-regulated [82]. To verify the best ratio in compatibility program, Chen et al. built a mouse cough model by citrate stimulation method with the cough time and the period recorded. The results found that the best ratio of Kuxingren (Armeniacae Semen) and Jiegeng (Radix Platycodi) was 1:1 [6]. Hebei Medical University and Nanjing University of TCM confirmed that Lianhua Qingwen Capsule can promote phenol red excretion in mice trachea, prolong cough latency and reduce cough times in ammonia water-induced cough mouse and citric acid-induced cough in a guinea pig model, exhibiting a significant anti-cough-phlegm effect.

### Resolving phlegm

In TCM theory, the movement of fluid in the body depends on the transportation of the spleen, the body fluid passages regulation of the lung, and the *qi* transformation of the kidney. The spleen, lung, and kidney are interdependent with each other for regulating body fluid. SARS-CoV-2 mainly invades the lung, leading to the loss of governing the regulation of body fluid passages, resulting in the formation of phlegm retention. Therefore, the important direction of the treatment for COVID-19 is to use expectorant drugs. Researchers developed several models for phlegm retention and most of them use the method of phenol red excretion from the trachea to observe its resolving phlegm effect. Using this model, Jinhuang Zhike Granule showed an effect of resolving phlegm [83].

It was believed that abnormal body fluid in the lung can be changed into phlegm retention, and Mahuang (Ephedrae Herba) has an effect of ventilating lung *qi* for diuresis [84]. In the prescription of wind-cold attack on the lung, Mahuang and other drugs play the role of relieving exterior syndrome, ventilating the lung, and relieving cough and asthma [85]. Mahuang belongs to the type of herbs warming the lung, which can be used to resolve fluid retention in chronic obstructive pulmonary disease [86]. Mahuang, the principal drug in the formula for the treatment of pediatric asthma, has the functions of dissipating phlegm and eliminating blood stasis, relieving cough and asthma [87]. The combination of Mahuang and Shegan (Belamcandae Rhizoma) could strengthen the function of resolving phlegm [88]. Xu et al. summarized the action of Shigao (Gypsum Fibrosum) in Mufangji Decoction and found that Shigao in Mufangji Decoction is the major active component in resolving phlegm and resolving masses [89].

The effect of the volatile oil of patchouli on resolving phlegm and relieving cough was studied. In the phenol red excretion in mice trachea experiment, it was found that the volatile oil of patchouli had a significant effect in resolving phlegm [75]. Pharmacological studies were conducted on the phlegm-resolving effects of volatile oil and water extract of patchouli respectively. The results showed that not only the volatile oil of patchouli had phlegm-resolving effects, but also the water extract had the similar effects, suggesting that more than one therapeutic chemical constituent existing in patchouli [39].

Li et al. analyzed and summarized the rules for the use of TCM in the treatment of severe pneumonia in children, and found that the most commonly used herb was Gancao, which showed the effect in relieving cough, removing phlegm and relieving asthma [90]. It was believed that Gancao in Zhigancao Decoction, governing the cold and heat pathogen of *zangfu* organs, could clear the heat, remove toxicity, dissipate phlegm, and relieve cough [91].

Xuebijing Injection could promote blood circulation, eliminate phlegm and remove stasis, which leads to its ability of treating chronic obstructive pulmonary disease [92]. COVID-19 belongs to the syndrome of dampness toxin pestilence, resulting in dampness transformed into phlegm retention. Qingfei Paidu Decoction showed detoxification, lung-clearing heat, and expectorant effects, as was systemically demonstrated in clinical practice [93]. In the treatment of influenza A (H1N1), the accumulation of phlegm-heat in the lung can be treated with tamiflu and Lianhua Qingwen Capsule in combination [94]. Lianhua Qingwen Capsule could also significantly improve the symptom of phlegm retention in chronic obstructive pulmonary disease [95].

## Conclusion and perspectives

The COVID-19 pandemic continues to spread globally and there is no wonder drug to effectively treat the disease as yet. It will still take a long time to develop new drugs although with efficient global cooperation. By summarizing the experiences accumulated from clinical practice, we found that TCM can provide sustaining power in the fight against the pandemic.

TCM prescriptions, based on the thoughts of syndrome differentiation and treatment, on the foundation of the classic prescriptions, adds or subtracts type or dosage of TCM in consideration of symptoms, reflecting the essence of TCM personalized treatment, such that different individual with different syndromes in different stages receives the most appropriate treatment. In this critical situation, TCM prescriptions based on the classic formulations and modified flexibly in consideration of the special situations of individual patients, may effectively cope with the frustration of our society in dealing with this new disease.

Through the accumulation of clinical experiences and evaluation of therapeutic effects of TCM on COVID-19, the “Three TCM prescriptions and three medicines” is developed and becomes a powerful weapon against COVID-19. The “Three TCM prescriptions and three medicines” all include the composition of Maxing Ganshi decoction (MXGSD) except Xubijing injection. Therefore, MXGSD should be in accordance with the central pathogenesis of TCM on COVID-19. At present, the characteristics of central TCM pathogenesis are dampness, toxin, heat and stasis from the period of disease onset to critical status [96]. MXGSD includes four herbal medicines, namely Mahuang, Kuxingren, Gancao and Shigao. From the view of TCM theory, Mahuang and Kuxingren contribute to dispelling exterior pathogen, removing dampness through diuresis, and ventilating lung *qi*; Shigao can clear heat pathogen and detoxification; Gancao regulates multiple herbal medicines properties and gastrointestinal tract function. Additionally, Xuebijing injection with the action of promoting blood circulation can target blood stasis in the pathology of COVID-19. Therefore, MXGSD and Xuebijing injection are able to attenuate the pathological evolution of COVID-19.

Network pharmacology of MXGSD suggests that quercetin, kaempferol, wogonin, naringenin and isorhamnetin are the 5 main active compounds associated with virus infection, inflammatory response and immunomodulatory signaling pathway in severe COVID-19 [97]. In the construction of a drug-component-target-disease network, 9 compounds (e.g. quercetin, kaempferol, naringenin and luteolin) played a key role in the entire network for anti-inflammatory, antiviral and immune-regulatory effects [98]. In the evaluation of MXGSD on cytokine storm of COVID-19, the results suggested that MXGSD increased T cell differentiation and homeostatic proliferation against virus replication, as well as negatively modulated inflammatory factors such as interleukin, TNF and integrin to attenuate cytokine storm [99]. Presently, although the life cycle of SARS-CoV-2 is not clear, ACE2 has been considered as the receptor for the SARS-CoV-2 viral entry. Network pharmacology analysis and molecular docking suggest that patchouli alcohol derived from Pogostemonis herba, tussilagone derived from Farfarae flos, ergosterol derived from Polyporus, asarinin derived from Asari Radix et Rhizoma, ephedrine hydrochloride derived from Ephedrae Herba, and shionone derived from Asteris Radix et Rhizoma in Qingfei Paidu decoction can bind to ACE2 to block SARS-CoV-2 from invading into host cells, and 232 of the QFPDT’s 790 putative targets are co-expressed with ACE2 [28]. In the analysis on the components of Xuebijing injection, it is showed that 22 compounds correspond to 70 proteins intersected with targets co-expressed with ACE2 [100]. However, ACE2 negatively modulates severity of lung edema and acute lung failure, downregulation of ACE2 can exacerbate lung pathologies [101]. Therefore, directly blocking ACE2 may not be the main therapeutic mechanism for TCM treating COVID-19. Moreover, numerous of Chinese medical foumulae are used via oral administration, if the active compounds (e.g. quercetin, kaempferol and luteolin) in the peripheral blood exerting effects needs to be further evidenced by experiments instead of network pharmacology or molecular docking. Based on the analysis on TCM pathologenesis and main symptoms (e.g. low fever, dry cough, myalgia, fatigue and diarrhea), it is believed that the pathogens of COVID-19 are related to dampness and toxin. In TCM theory, dampness can directly injure gastrointestinal tract function, so the symptoms of nausea, vomit and diarrhea become common. Research reported SARS-CoV-2 RNA was found in the feces of infected patients, and the intestinal epithelial cells also express ACE2 receptors. The phenomenon points out the role that the gut microbiota may play in COVID-19, and ameliorating gut microbiota profile by personalized nutrition and supplementation for regulating immunity should be an appropriate prophylactic and therapeutic approaches [102]. Additionally, a clinical trial showed that the gut microbial profile of 30 patients with COVID-19 was different from that of H1N1 patients and health persons [103]. Modulation of Gut-Lung axis can directly influence the immune function of the lung, e.g. probiotics, prebiotic dietary fiber and other plant nutritional bioactives can attenuate COVID-19 or its associated symptoms [104–106]. Based on the above opinions, we deduce that the mechanisms of the “Three TCM prescriptions and three medicines” are associated with regulation of gut microbial profile, and these oral formulae regulate immune responses by Gut-Lung axis against COVID-19. As the key composition of the “Three TCM prescriptions and three medicines”, MXGSD not only increases the abundance of intestinal microbiota related to the production of short-chain fatty acids in nomral mice, but also reduces the pathogenic bacteria Tyzzerella and Treponema in intestinal flora of mice with influenza by High-throughput Sequencing [107, 108]. Study also demonstrated that Xuebijing Injection increased the content of beneficial bacteria, improved the intestinal mucosal barrier function, and reduced the release of endotoxin and pro-inflammatory factors in severe acute pancreatitis rats [109]. Focusing on the regulation of Gut-Lung axis and amelioration of homeostasis of intestinal microenvironment perhaps is the mechanism of the “Three TCM prescriptions and three medicines” for the treatment of COVID-19.

TCM has played and will continue to play an important role in the fight against the coronavirus pandemic, which is bound to increase more attention and application of TCM in China and abroad. We believe that the prescriptions with the advantages of syndrome differentiation and treatment, as well as the convenience of use and high therapeutic effects and safety features, can make TCM shine on the world stage and better serve human health.

## Data Availability

Not applicable.

## Abbreviations

COVID-19: Coronavirus disease 2019
TCM: Traditional Chinese medicine
ACE2: Angiotensin-converting enzyme 2
PTGS2: Prostaglandin-endoperoxide synthase 2
BCL2: B-cell lymphoma-2
CASP3: Caspase 3
HSP90AB1: Heat shock protein 90 kDa alpha (cytosolic), class B member 1
HSP90AA1: Heat shock protein 90 kDa alpha A1
PTGS1: Prostaglandin-endoperoxide synthase 1
NCOA2: Nuclear receptor coactivator 2
SARS-CoV-2 3CL: Severe acute respiratory syndrome coronavirus 2 3C-like
H1N1: Hemagglutinin 1 neuraminidase 1
JAK-STAT: Janus-activated kinase Singal transducers and activators of transcriprion
TNF-α: Tumor necrosis factor-α
IL-6: Interleukin-6
ErbB: Erythroblastic leukemia viral oncogene homolog
CT: Computed tomography
TLR4: Toll-like receptor 4
TLR7: Toll-like receptor 7
MyD88: Myeloid differentiation factor 88
TRAF6: Tumor necrosis factor receptor-associated factor 6
sICAM-1: Soluble inter cellular adhesion molecule-1
Th1: Helper T 1 cell
Th2: Helper T 2 cell
INF-γ: Interferon-γ
IL-4: Interleukin-4
TGFB1: Transforming growth factor beta-1
NFKB2: Nuclear factor kappa B subunit 2
MMP9: Matrix metalloprotein-9
NR3C1: Nuclear receptor subfamily 3 group C member 1
PGR: Plant growth regulator
CACC1: Calcium-activatied chloride channel 1
CACC4: Calcium-activatied chloride channel 4
TMEM16A: Transmem-brane protein 16A
